# The replication stress response and the ubiquitin system: a new link in maintaining genomic integrity

**DOI:** 10.1186/1747-1028-5-8

**Published:** 2010-03-10

**Authors:** Deanna M Koepp

**Affiliations:** 1 Department of Genetics, Cell Biology and Development, University of Minnesota, Minneapolis, MN 55455, USA

## Abstract

Maintenance of genomic integrity is important for cellular viability and proliferation. During DNA replication, cells respond to replication stress by activating checkpoint pathways that stabilize replication forks and prevent cell cycle progression. The *Saccharomyces cerevisiae *F-box protein Dia2 is a ubiquitin ligase component required for genomic stability and may help replication complexes negotiate damaged DNA or natural fragile sites. We recently implicated Dia2 in the replication stress response. We demonstrated that Dia2 is targeted for ubiquitin-mediated proteolysis and that activation of the S-phase checkpoint inhibits Dia2 protein turnover. S-phase checkpoint mutants fail to stabilize the Dia2 protein and checkpoint mutants that lack Dia2 exhibit increased sensitivity to replication stress. We also showed that Dia2 protein turnover is not the result of an autocatalytic mechanism. Instead, an N-terminal 20 amino acid motif that is also required for nuclear localization is necessary for Dia2 proteolysis. Dia2 mutants lacking this motif but modified with an exogenous strong nuclear localization signal are both nuclear and stable and disrupt cell cycle dynamics. In summary, our studies suggest that inhibition of Dia2 proteolysis is a novel target of the S-phase checkpoint. We think that this work will help to identify the mechanisms that function downstream of checkpoint activation and that intersect with cell cycle control pathways.

## Introduction

Accurate DNA replication is critical to faithful chromosome segregation and cell viability. The progression of DNA synthesis may be hindered by the presence of damaged DNA or genotoxic stresses, which can lead to replication fork stalling or fork collapse. Such events have the potential to induce genomic instability, one of the hallmarks of cancer cells. To maintain genomic stability, checkpoint responses are activated during replication stress or in response to defects during DNA replication (reviewed in [1,2]). Activation of checkpoint pathways promotes cell viability by stabilizing replication forks, suppressing the firing of late replication origins and inhibiting the progression of S phase [3-7]. By delaying further progress through S phase, checkpoint activation allows time for the replication defects to be resolved, thus preventing damage to chromosomes.

Checkpoint mechanisms are often defective in cancer cells, suggesting that the absence of these surveillance pathways contributes to a proliferative advantage in tumor formation (reviewed in [8-11]). Therefore, determining how checkpoint mechanisms intersect with DNA replication and cellular proliferation pathways has implications for understanding cancer biology. There has been remarkable progress in identifying key proteins required for activation of checkpoint signaling pathways [1,2], but little is known about the molecular targets of these pathways and how they inhibit progression through S phase. The goal of this commentary is to describe recent work from my laboratory [12] that suggests the proteolytic regulation of the budding yeast ubiquitin ligase component Dia2, a protein previously determined to be required for genomic stability [13-15], is a target of the replication checkpoint pathway.

## Discussion

### Dia2, DNA replication, and genomic stability

Dia2 functions as a ubiquitin ligase specificity adaptor and is required for genomic stability [13,14]. In the absence of Dia2, cells are hypersensitive to DNA damaging agents, accumulate DNA damage foci and exhibit increased chromosome loss and rearrangements, particularly at repetitive regions such as the rDNA locus [13-15]. The replication checkpoint is constitutively active in *dia2 *null cells, as evidenced by hyperphosphorylation of the checkpoint kinase Rad53 [14,15]. The *dia2 *deletion strain shows synthetic interactions with a number of mutants in DNA replication and checkpoint proteins [13-16]. Together, these phenotypes indicate that Dia2 is important to maintain a stable genome and that it is likely to have a role in DNA replication or the replication stress response. To better understand the role of Dia2, we undertook a study examining the proteolytic regulation of the Dia2 protein.

### Proteolytic regulation of F-box proteins

SCF complexes are multi-component ubiquitin ligases that function in many cellular processes, including cell cycle control and signaling pathways (reviewed in [17,18]). F-box proteins function as specificity adaptors for SCF ubiquitin ligases by binding both the substrate protein to be ubiquitinated and interacting with the rest of the complex [19,20]. The conserved F-box domain is required to bind the Skp1 component in the complex [19,20]. There are many F-box proteins; thus there are many individual SCF complexes and the specificity of each is determined by which F-box protein is bound. A convenient way to regulate the activity of SCF ubiquitin ligases, therefore, is to control the availability of the F-box protein.

Most F-box proteins are found at lower abundance than the rest of the SCF complex components. This is often accomplished by proteolytic regulation of the F-box protein itself [21,22]. One major mechanism is that of autoubiquitination, in which the F-box protein is tagged with ubiquitin and targeted for destruction by interacting with the SCF core complex in the absence of a substrate protein. This mechanism has been proposed to be a means to keep SCF ubiquitin ligase activity in check when the substrate protein is not available [21-23]. *In vitro*, many F-box proteins can serve as substrates in autoubiquitination reactions [24], but the extent to which this process occurs *in vivo *is unclear. By contrast, a few F-box proteins are destabilized when SCF components are impaired [25-28]. Moreover, some F-box proteins are targeted for degradation by SCF-independent pathways. For example, the human F-box protein Skp2 is a cell cycle-dependent substrate of the APC ubiquitin ligase [29].

We observed that Dia2 is an unstable protein, targeted for degradation by the ubiquitin proteasome system, in a cell cycle dependent manner. We demonstrated that the abundance of the Dia2 protein is low in G1, but then increases as cells enter S phase [12], consistent with a role for Dia2 in DNA replication or the replication stress response. To determine whether Dia2 protein turnover was the result of an autoubiquitination mechanism, we examined the half-life of the Dia2 protein in *scf *mutants [12]. We found that Dia2 was not stabilized in *scf *mutants. Instead, Dia2 appeared even less stable in *skp1 *and *cdc53 *mutants, consistent with the possibility that an intact SCF complex promotes Dia2 stability. In addition, we showed that a Dia2 mutant lacking the F-box domain has the same half-life as wildtype Dia2 [12]. Together, these data indicate that Dia2 proteolysis is not controlled by an autoubiquitination mechanism.

A standard structure-function approach was used to determine which domain in Dia2 is required for its turnover. We demonstrated that a 20 amino acid motif in the N-terminal TPR repeat region of Dia2 is required for its proteolysis [12]. Remarkably, we found that this same motif is required for localization of Dia2 to the nucleus. The addition of a strong NLS to Dia2 mutants lacking the 20 amino acid motif led to stable, nuclear forms of Dia2. Overexpression of these stable, nuclear forms of Dia2 disrupted cell cycle progression, leading to an accumulation of cells in G1 phase with a concomitant decrease in G2/M phase cells [12]. These results suggest that failure to degrade the Dia2 protein interferes with cell cycle control.

### Activation of the replication checkpoint inhibits Dia2 proteolysis

To explore the cell cycle dynamics of Dia2 protein degradation, the stability of Dia2 was determined in cells arrested in G1, S and G2/M phases of the cell cycle. We showed that the Dia2 protein is most unstable in G1, but is significantly stabilized in cells arrested in S phase [12]. Since the S-phase arrest relies on activation of the S-phase checkpoint, we examined whether checkpoint activation was required for stabilization of Dia2 by analyzing the turnover of the Dia2 protein in a number of checkpoint mutants. Strikingly, we found that the Dia2 protein was unstable when the S-phase checkpoint was defective [12]. This result suggests that inhibiting Dia2 protein turnover might be a functional consequence of activation of the S-phase checkpoint.

We reasoned that if Dia2 protein levels are regulated by the S-phase checkpoint, then removing Dia2 from checkpoint mutants should exacerbate the phenotypes of checkpoint mutants. We examined a series of *dia2Δ *checkpoint double mutants for their sensitivity to hydroxyurea, which induces replication stress. These double mutants exhibited significantly increased sensitivity to replication stress under these conditions [12], consistent with the hypothesis that Dia2 protein turnover is required for the cellular response to replication stress.

## Conclusions

**Figure 1:**
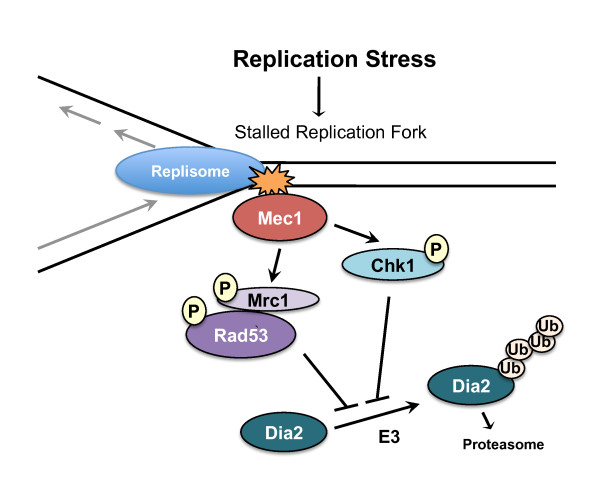
**Model for S-phase checkpoint regulation of Dia2 ubiquitin-mediated destruction**. In response to replication stress, replication forks stall and activate S-phase checkpoint pathways, leading to the inhibition of Dia2 protein turnover. Our data suggest that Dia2 stabilization is downstream of both Rad53 and Chk1 activation and work from other groups indicate that Dia2 binds to Mrc1 [31,32].

We demonstrated that activation of the S-phase checkpoint inhibits Dia2 protein degradation and we propose that SCF^Dia2 ^activity is required for the replication stress response (see model in Figure 1). Previous work had suggested that Dia2 is required to help replication complexes traverse areas of the genome that are difficult to replicate and prone to DNA damage. Our studies suggest that as replication complexes encounter difficulties and activate the checkpoint, Dia2 is stabilized, presumably to form an active SCF^Dia2 ^complex. This model predicts that Dia2 might interact with components of the checkpoint pathway. Consistent with this hypothesis, recent studies have determined that Dia2 associates with replisome complexes [30-32]. Strikingly, Dia2 binds to Mrc1 [31,32], a replication protein that also has a critical role in activation of the S-phase checkpoint [3,7]. Mrc1 is important for activation of the Rad53 checkpoint effector kinase [3,7]. Our results suggest that stabilization of Dia2 occurs downstream of Rad53 activation. Altogether, the data point to Dia2 protein stabilization as a target of the S-phase checkpoint.

How Dia2 protein turnover is controlled by the S-phase checkpoint is an open question. Two straightforward possibilities are: 1) that activation of the checkpoint pathway prevents Dia2 from being targeted for ubiquitin-dependent proteolysis or 2) that the ubiquitination pathway that controls Dia2 protein turnover may be inactivated when the S-phase checkpoint is triggered. Our study did not address the identity of the pathway responsible for Dia2 protein degradation, so future work will be necessary to distinguish between these two possibilities.

Our work adds to the number of ubiquitin ligase components that have been shown to be regulated by ubiquitin-mediated proteolysis. For Dia2, we demonstrated that this regulation is not the result of autoubiquitination, but rather the activity of an independent pathway. It is intriguing that the 20-amino acid motif that is responsible for Dia2 protein instability is also critical for its nuclear localization. Nuclear localization signals are rich in positively-charged amino acids such as lysine residues, which also serve as the acceptor sites for ubiquitin modification. This may simply be an interesting coincidence, but it is tempting to speculate that the pathways responsible for ubiquitination and nuclear localization of the Dia2 protein may counteract each other.

By studying the proteolytic regulation of a ubiquitin ligase component, we have identified a novel target of the S-phase checkpoint. We think that this work serves as a starting point to elucidate the cellular pathways and key players that function downstream of checkpoint activation and intersect with cell cycle control mechanisms. We anticipate that determining the molecular mechanisms involved in checkpoint-mediated cell cycle inhibition is likely to expand our understanding of tumor biology, as checkpoints are frequently defective in cancer cells.

## Competing interests

The author declares that they have no competing interests.

## Authors' contributions

D.M.K. devised and wrote the manuscript.
